# A Novel Quantum-Behaved Bat Algorithm with Mean Best Position Directed for Numerical Optimization

**DOI:** 10.1155/2016/6097484

**Published:** 2016-05-18

**Authors:** Binglian Zhu, Wenyong Zhu, Zijuan Liu, Qingyan Duan, Long Cao

**Affiliations:** ^1^Communications Engineering, Chongqing University, Chongqing 400030, China; ^2^Jiuquan Satellite Launch Center, Jiuquan 732750, China

## Abstract

This paper proposes a novel quantum-behaved bat algorithm with the direction of mean best position (QMBA). In QMBA, the position of each bat is mainly updated by the current optimal solution in the early stage of searching and in the late search it also depends on the mean best position which can enhance the convergence speed of the algorithm. During the process of searching, quantum behavior of bats is introduced which is beneficial to jump out of local optimal solution and make the quantum-behaved bats not easily fall into local optimal solution, and it has better ability to adapt complex environment. Meanwhile, QMBA makes good use of statistical information of best position which bats had experienced to generate better quality solutions. This approach not only inherits the characteristic of quick convergence, simplicity, and easy implementation of original bat algorithm, but also increases the diversity of population and improves the accuracy of solution. Twenty-four benchmark test functions are tested and compared with other variant bat algorithms for numerical optimization the simulation results show that this approach is simple and efficient and can achieve a more accurate solution.

## 1. Introduction

In recent years, with the need of optimization problems in reality, all kinds of bioinspired optimization algorithms or swarm intelligence optimization algorithms have been proposed, such as the genetic algorithm (GA) [1], differential evolution (DE) [2], ant colony optimization (ACO) [3], firefly algorithm (FA) [4, 5], cuckoo search (CS) algorithm [6, 7], particle swarm optimization (PSO) [8], artificial bee colony (ABC) optimization [9], and bat algorithm (BA) [10, 11]. These bionic intelligent algorithms are random search methods which mimic natural biological systems [12]; entirely instinct depends on the organism itself, by the unconscious optimization behavior to optimize its survival to adapt to the environment. Compared with the traditional optimal algorithms, they do not depend on the characteristic of strict mathematical optimization problem itself and have the characteristic of strong parallelism. Each individual has self-organization and evolution; as a result, some high dimensional optimization problems are superior to the traditional methods.

The bat algorithm is a new metaheuristic method which was proposed by Yang in 2010 [10, 11]. The capability of echolocation of microbats is fascinating as these bats can find their prey and discriminate different types of insects even in complete darkness [13]. Inspired by the echolocative behavior of bats, this algorithm carries the search process using artificial bats as search agents mimicking the natural pulse loudness and emission rate of real bats. When these bats are chasing prey, they tend to decrease the loudness and increase the rate of pulse emission. Bat algorithm has a good optimization performance in low dimensional case [14–16] and is widely used in engineering optimization [17, 18] and multiobjective optimization [19]; however, due to low population diversity, it severely suffers from premature convergence problem in the high dimensional case [20]. So many variant bat algorithms are proposed to enhance the population diversity and to avoid being trapped into local optimum. In [21], Lin et al. put forward a chaotic Levy flight bat algorithm (CLBA) for parameter estimation in nonlinear dynamic biological system; in [13], Xie et al. introduced the difference operator and Levy flight trajectory into the bat algorithm (DLBA) to solve function optimization problems; in [22], Yılmaz and Kucuksille, inspired by standard particle swarm optimization (PSO) [8] algorithm and artificial bee colony (ABC) algorithm [9], proposed improved bat algorithm (IBA). In IBA, the velocity of each bat is updated with linear decreasing inertia weight factor and the frequency is self-adaptive to improve the exploration and exploitation. In [23], Wang and Guo put the harmony search method into the bat algorithm and developed a hybrid metaheuristic HSBA method for optimization problem, speeding up convergence of bat algorithm; in [24], Yılmaz et al. have studied the mechanism of updating loudness and pulse emission rate of BA and they found that loudness and pulse emission rate provide a balance between exploitation and exploration. So they modify the equations to improve the exploration capability of BA. Afrabandpey et al. [25] introduced the chaotic sequences into bat algorithm in different ways to avoid premature convergence. In [26], four parameters of BA are replaced by the chaotic maps separately; meanwhile, in [20], loudness and pulse emission rate are tuned via multiplying a linear decreasing or increasing function by a chaotic map function; in [25], chaotic map takes place of random number for parameter initialization. These approaches, to some extent, can avoid getting trapped into local minimum. However, in QMBA, bats adopt different search strategies in different times and have the mechanism of jumping out of local optimal solution; these strategies enhance the convergence speed of the algorithm and improve the accuracy of solutions.

The rest of paper is organized as follows.  Section 2 describes the standard BA and Section 3 presents the quantum-behaved bat algorithm with the direction of mean best position. The simulation and comparison of this proposed algorithm are presented in Section 4. Finally, general conclusions are drawn in Section 5.

## 2. The Bat Algorithm

Echolocation is a very important character of bats; Yang proposed bat algorithm by mimics of bats' foraging behavior. Bats fly randomly in the air or in the process of searching for prey by using echolocation to catch food and to avoid obstacles. In order to transform these behaviors of bats to algorithm, there are some approximations and idealized rules [10].

(i) All bats use echolocation to sense distance, and they also “know” the difference between food/prey and background barriers in some magical way.
(ii) Bats fly randomly with velocity *v*_*i*_ at position *x*_*i*_ with a fixed frequency *f*_min_, varying wavelength *λ*, and loudness *A*_0_ to search for prey. They can automatically adjust the wavelength (or frequency) of their emitted pulses and adjust the rate of pulse emission *r* ∈ [0,1], depending on the proximity of their target.
(iii) Although the loudness can vary in many ways, we assume that the loudness varies from a large (positive) *A*_0_ to a minimum constant value *A*_min_.

In the BA, for the *i*th bats of swarm having position **x**_*i*_ (solution), velocity **v**_*i*_, and frequency *f*_*i*_, each bat will move toward the current best position (solution), and its position, velocity, and frequency are updated during the course of iteration as follows:

(1) fi=fmin+fmax−fminβ,vit=vit−1+xit−1−xgt−1fi,xit=xit−1+vit,

where *β* is a random number of a uniform distribution in [0,1] and **x**_*g*_^*t*−1^ represents the current global best solution (position) after comparing all the solutions (positions) among all the *n* bats. These equations can guarantee the exploration ability of BA.

For the local search, when a solution is selected among the current best solutions, a new candidate solution can be generated as

(2) $$\begin{array}{c} x_{new}=x_{old}+\varepsilon {\overset{-}{A}}^{t}, \end{array}$$

where *ε* is a random number in [−1,1] and directs new solution apart from or close to the current best solution. Here, ${\overset{-}{A}}^{t}$ is mean value of all bats of loudness.

When finding prey, bat will gradually decrease the loudness and increase the rate of pulse emission in order to track its prey to capture it. The loudness and pulse emission rate update accordingly as the iterations proceed as shown in

(3) Ait=αAit−1,rit=ri01−exp⁡−γt,

where *α* and *γ* are constants. In fact, the *α* parameter controls the convergence of bat algorithm and therefore plays a similar role as the cooling factor in the simulated annealing algorithm [27]. For simplicity, we set *α* = *γ* = 0.9 in our simulations.

The basic steps of BA can be summarized as the pseudocode shown in Algorithm 1.

## 3. Quantum-Behaved Bat Algorithm with the Direction of Mean Best Position

The standard bat algorithm has quick convergence and is easy to be implemented; therefore, it has been widely applied in practical engineering. However, BA is more easily to fall into local optimal point when optimizing the multimodal functions. There are many reasons for prematuration of BA. Firstly, through the analysis of the trajectory of bats, we found that many bats are trapped into local optimal point because of decreasing diversity of bats. Secondly, other bats are directed only by current optimal solution, if only the best bat falls into local point and it will misguide others.

Thirdly, there are no mechanisms to jump out of local optima in BA. In order to solve the above problem, the behavior of quantum of bats is introduced into the algorithm to increase the diversity of population and it also contributes to avoiding prematuration. The current global optimal solution is used to guide other bats flying in the early stage of searching, while mean best position is used in later stage of searching. That can enhance the efficiency and convergence speed of BA.

Our proposed algorithm (QMBA) is based on the basics of framework of original bat algorithm. The parameters *r* and *A* control the exploitation and exploration, respectively, by updating the two factors to guide BA into local search or global search; however, the new candidate solutions are generated by the following formulas which are different from original BA:

(4) $$\begin{array}{c} x_{i,j}^{t}=\left\{\begin{array}{cc} x_{i,j}^{t-1}+\left(x_{j}^{*}-x_{i,j}^{t-1}\right)rand, & \delta_{j}>TH,\\ x_{i,j}^{t-1}+\varepsilon , & \delta_{j}\leq TH, \end{array}\right. \end{array}$$

where

(5) $$\begin{array}{c} \delta_{j}=\left|\;x_{j}^{*}-x_{i,j}^{t-1}\;\right|, \end{array}$$

and it indicates the distance between the position of *j*th dimension of *i*th bat and the *j*th dimension of current optimal position among all bats, and rand is a random number in [0,1]. If *δ*_*j*_ is greater than the threshold TH, it means the distance between *i*th bat and current optimal position is far; hence, the current bat moves toward optimal position so far by random step. However, if *δ*_*j*_ is less than the threshold TH, it suggests that the current bat is nearby the current optimal position; therefore, the bat flies randomly. The diversity of bat population and the exploration ability are improved by self-adapting the step in terms of distance.

During the process of search, according to certain probability of mutation *p*_*m*_, some bats will be mutated with quantum behavior [28, 29]; these bats are updated with the following formulas:

(6) xit=xbestt+αMbestt−xit−1ln1rand,rand>0.5,xbestt−αMbestt−xit−1ln1rand,rand≤0.5,

where *α* is the contraction-expansion coefficient defined as

(7) $$\begin{array}{c} \alpha =\alpha_{0}-\frac{\alpha_{0}-\alpha_{1}}{T}t. \end{array}$$

*α*_0_ and *α*_1_ are the initial and the final values of *α*, respectively, and *T* is the maximum number of cycles. We usually set *α*_0_ = 1 and *α*_1_ = 0.5 to obtain good performance in general [30].

*M*_best_^*t*^ is the mean best position defined as the average of *P*_*i*_^*t*^ positions of all bats. That is,

(8) $$\begin{array}{c} M_{best}^{t}=\frac{1}{M}\left(\;\overset{M}{\underset{i=1}{\sum }}P_{i,1}^{t},\overset{M}{\underset{i=1}{\sum }}P_{i,2}^{t},\overset{M}{\underset{i=1}{\sum }}P_{i,3}^{t},\ldots ,\overset{M}{\underset{i=1}{\sum }}P_{i,D}^{t}\;\right), \end{array}$$

where *P*_*i*_^*t*^ indicate the best position of the *i*th bat experienced, *M* is the population size, and *D* is the dimension of problem. Bat with quantum behavior increases the diversity of population and contributes to jumping out of the local optima.

In the late state of searching, positions of bats are updated as follows:

(9) $$\begin{array}{c} x_{i}^{t}=x_{i}^{t-1}+\left(\;M_{best}-x_{i}^{t-1}\;\right)*rand. \end{array}$$

The mean best position is used to guide other bats flying in the late stage of searching; it improves the accuracy of solutions and speeds up the convergence of the algorithm because of using the statistical information of better position of bats. The pseudocode of quantum-behaved bat algorithm with mean best position is shown in Algorithm 2.

## 4. The Simulations

In order to verify the efficiency of the proposed algorithm, we select twenty-four standard benchmark functions [24, 31, 32] to test the ability to search QMBA. The results are compared with BA and other variants of BA to show the performance of global numerical optimization. For this purpose, we have used BA, IBA, and HSBA to carry out numerical experiments for 24 standard test benchmarks, and the results will be discussed in this section.

### 4.1. Benchmark Functions

There are many different functions used to test the performance of algorithms. These benchmark functions can be divided into four categories. Category I is unimodal, category II is multimodal, category III is shifted or rotated, and category IV is composite function.  Table 1 lists these functions, respectively, where *D* indicates the dimension of the function, range is the boundary of the function's search space, and *f*_min_ is the minimum value of the function. Unimodal function (F1~F6) has only one extreme point and these algorithms can easily find the point; however, in multimodal function (F7~F12) there will be many local minimums; it is relatively difficult to find the global minimum. As the global minimum of most benchmark functions are zero for all dimensions, therefore, we select some shifted and rotated functions to test the algorithm's robustness. For shifted and rotated functions (F13~F18) which have different parameter values for different dimensions, as can be seen in Table 1, F13, F14, and F15 are shifted functions whose global optimum is shifted to random positions to make different parameter values for different dimensions, where **z**_*i*_ = **x**_*i*_ − **o**_*i*_, **o**_*i*_ define the new shifted optimum position for primitive test function. The other way is rotating the functions by using *F*(*x*) = *f*(*R∗x*) formula, where *R* is an orthogonal rotation matrix, such as F16, F17, and F18. Finally, composite test function (F19~F24) combines different other test functions by stretching and manipulating their coverage range. It should be noted that **M**_*i*_ define the linear transformation matrix for each *f*_*i*_(*x*), *σ*_*i*_ is used to control each *f*_*i*_(*x*)'s coverage range, and *λ*_*i*_ indicate the stretch or compression level of the primitive test functions (see [32] for the detailed description of the composite functions). These composite functions provide challenge to find the global optimum, but that can verify the searching capability of the algorithm effectively.

### 4.2. Parameter Setting

In order to determine whether QMBA algorithm can be as effective as BA and other variants of BA, we compared its performance on numerical optimization with BA and other variants of BA, which include BA with inertia weight (IBA) [22], modified bat algorithm (MBA) [24], BA with harmony search (HSBA) [23], and bat algorithm based on chaotic map (CBA) [25].

All these algorithms are tested with 30 independent runs, the number of bats in population is fixed to 50, the dimension of problem is 30, and maximum number of iterations is set to 900 except HSBA which is 300, so these algorithms have reached 45000 FEs. The other parameter settings of these algorithms are given in Table 2.

### 4.3. Comparison of Experiment Results

The comparisons of test results of BA and other variants of BA are shown in Tables 3, 5, 7, and 9. As can be seen from these tables, the mean fitness values, maximum values, minimum values, and standard deviations are obtained in thirty trails.

According to Derrac et al. [33], to improve the evaluation of evolutionary algorithms' performance, statistical tests should be conducted. In other words, it is not enough to compare algorithms based on the mean and standard deviation values. A statistical test is necessary to prove that a proposed new algorithm presents a significant improvement over other existing methods for a particular problem.

In order to judge whether the results of the QMBA differ from the best results of the other algorithms in a statistically significant way, a nonparametric statistical test, *t*-test [32, 33], was carried out at a 5% significance level. The *p* values calculated in *t*-test comparing QMBA and other algorithms over all the benchmark functions are given in Tables 4, 6, 8, and 10. In these tables, according to Derrac et al. [33], those *p* values that are less than 0.05 could be considered as strong evidence against the null hypothesis.

In the following subsections, the details of the results and discussion at each group of benchmark functions are provided.

Unimodal functions can evaluate the capability of exploitation of an optimization algorithm because they have only one global optimum. For function F1, Sphere Function, only QMBA could get a solution near the global optimal solution, and other algorithms are trapped into local minimum. F2 is Schwegel's problem 2.22. For this function, HSBA and QMBA outperform all the other algorithms; however, QMBA offered the highest accuracy of solutions. For function F3, Schwegel's problem 1.2, this function is difficult to optimize because there are too many terms for high dimensional ones, but the mean fitness value of QMBA was the best among all the algorithms. In the results for F4, QMBA performed significantly better than any other algorithm. The fifth function F5 is known as Rosenbrock function which has a very narrow valley from local optimum to global optimum. So the algorithms are easily trapped into local optimum. But QMBA obtained the best solution among all the algorithms and its advantages over the competitors were statistically significant. In results obtained for F6, QMBA offered the highest accuracy. As can be seen in Table 6, the *p* values indicate that QMBA achieves significant improvement in all the unimodal benchmark functions compared to other algorithms.

The results of the multimodal functions (F7–F12) are provided in Table 5. It should be noted that these benchmark functions have many local optimums with the number increasing exponentially with dimension, so they are useful for evaluating the exploration ability of an optimization algorithm. As observed in Tables 5 and 6, QMBA performs better than other algorithms in all benchmark multimodal functions. That means QMBA has also a very good exploration ability.

The results of shifted and rotated functions are provided in Tables 7 and 8. These functions can be used to test the algorithms' robustness. As the results of mean, standard deviation, and minimum and maximum show, QMBA performs better than the other algorithms in two of the shifted and rotated (F14, F16) functions. The HSBA has the best results in two, and IBA and MBA have only one in category III, separately. It should be noted that HSBA and MBA are not significantly better than QMBA in functions F13 and F15, respectively. So it can be claimed that the results of QMBA in these functions are slightly better than the other algorithms.

The results of hybrid composite benchmark functions are provided in Tables 9 and 10. For function F19, QMBA has the best results, but the *p* values of *t*-test in Table 10 show that the results of QMBA are not significantly better than HSBA. In the remaining functions (F22, F23, and F24), the results for QMBA are significantly better than the other algorithms except for functions F20 and F21. Therefore, the results can strongly show that the QMBA has high performance in dealing with complex problem as well.

**Figure 1:**
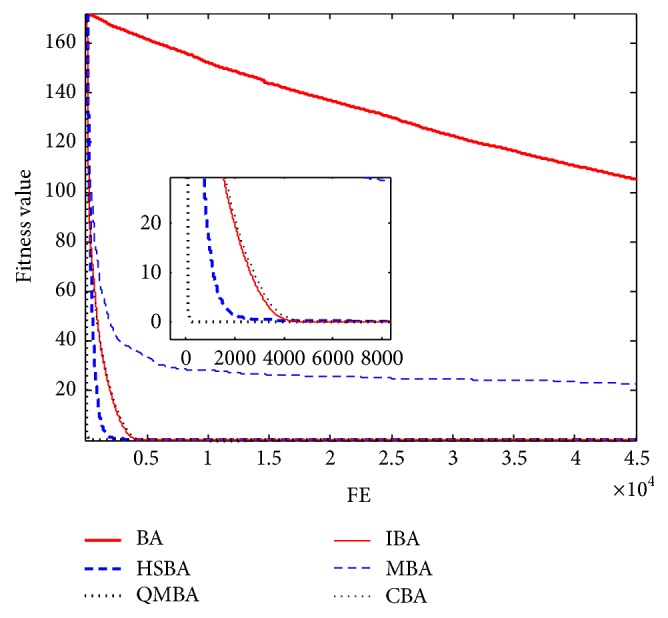
The curve of fitness value for F1.

**Figure 2 fig2:**
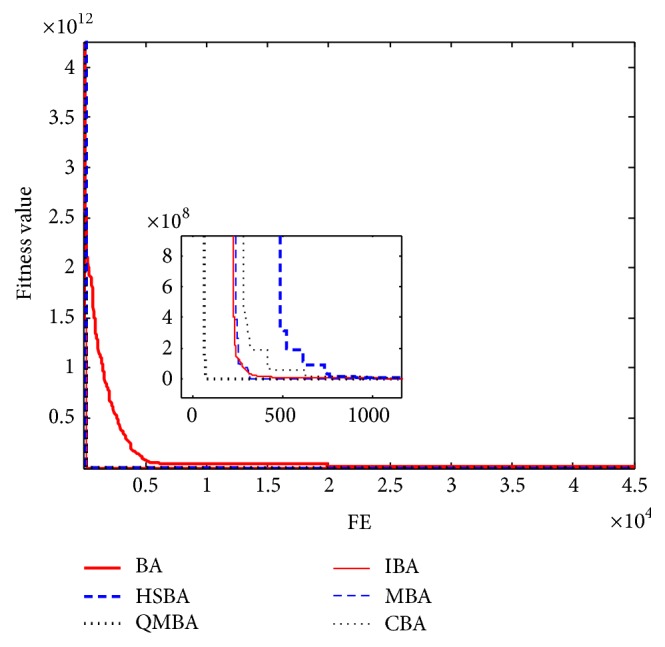
The curve of fitness value for F2.

**Figure 3 fig3:**
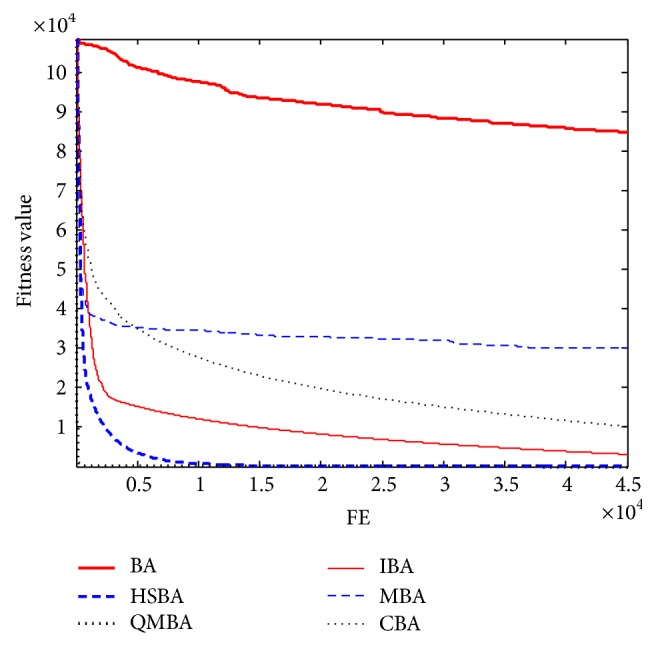
The curve of fitness value for F3.

**Figure 4 fig4:**
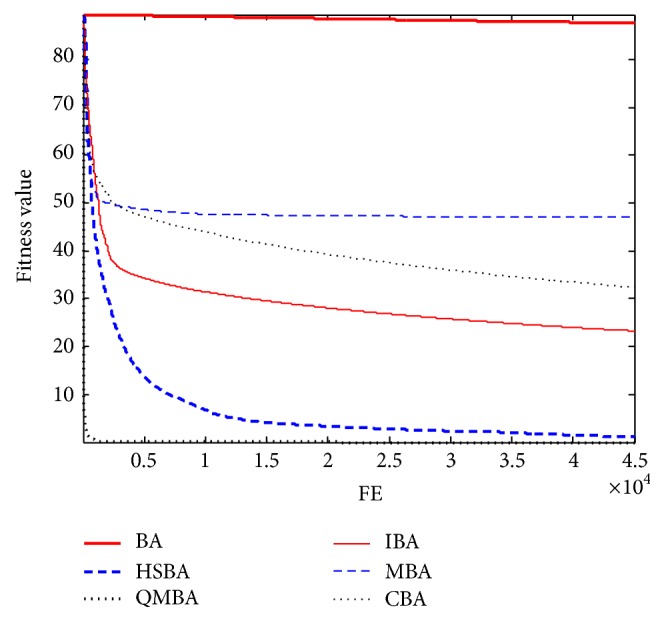
The curve of fitness value for F4.

**Figure 5 fig5:**
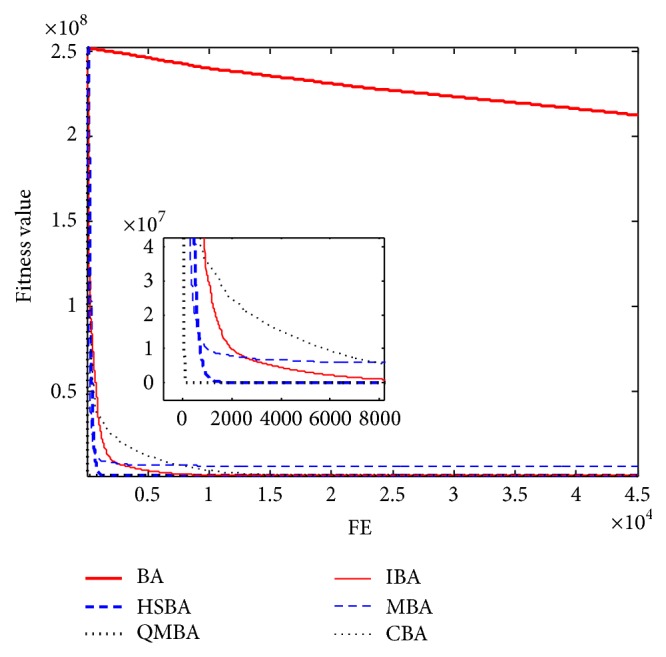
The curve of fitness value for F5.

**Figure 6 fig6:**
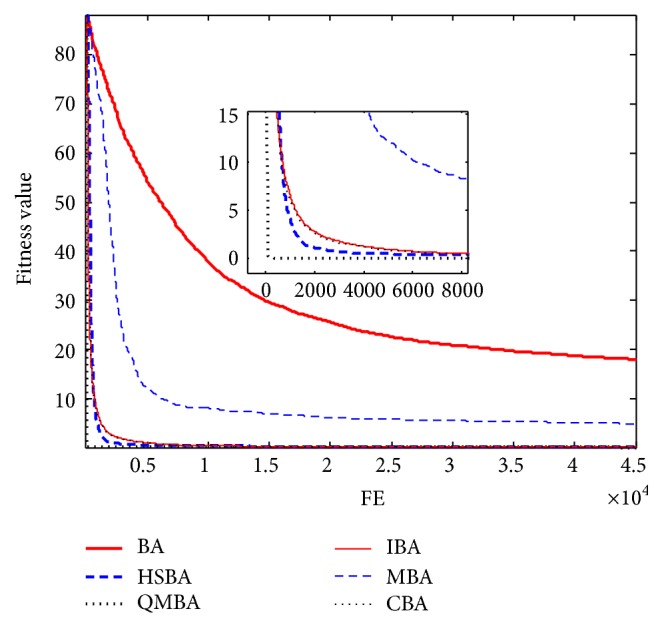
The curve of fitness value for F6.

**Figure 7 fig7:**
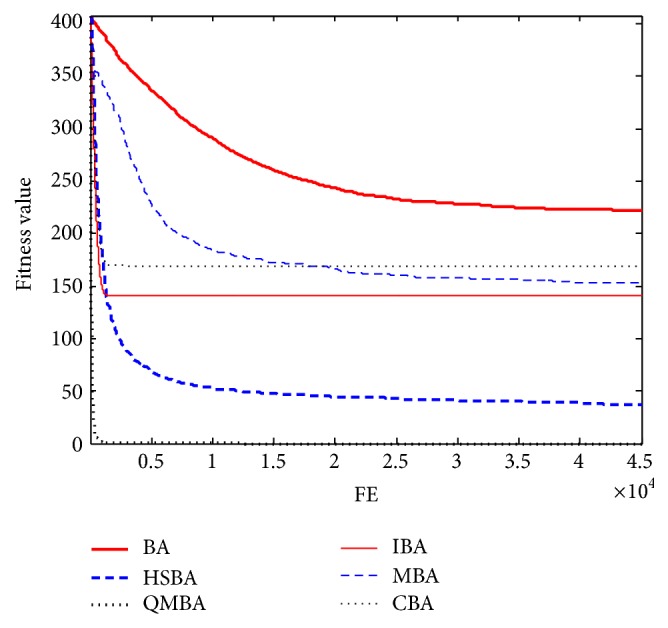
The curve of fitness value for F7.

**Figure 8 fig8:**
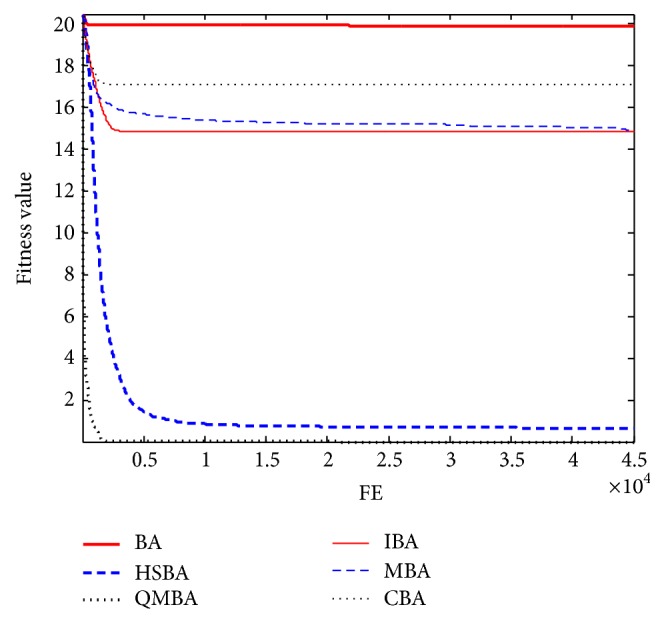
The curve of fitness value for F8.

**Figure 9 fig9:**
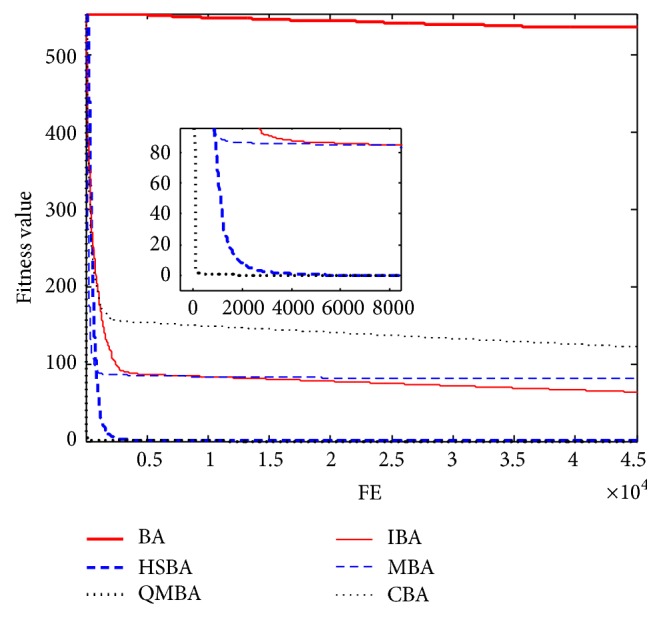
The curve of fitness value for F9.

**Figure 10 fig10:**
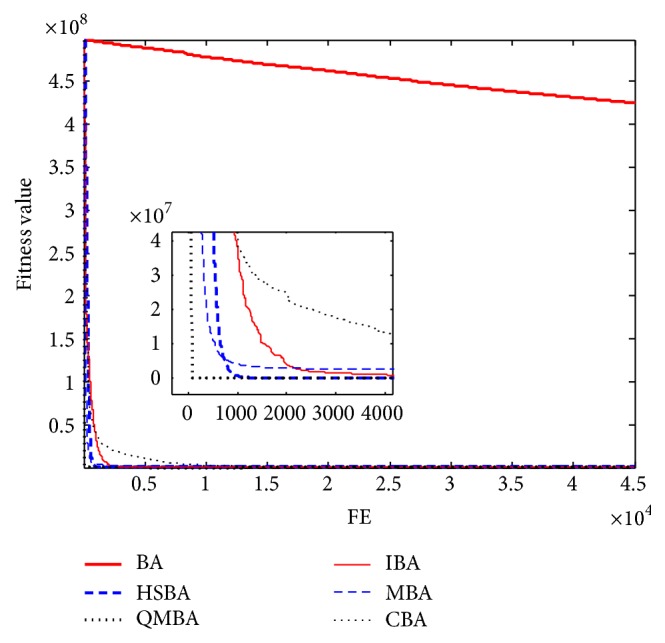
The curve of fitness value for F10.

**Figure 11 fig11:**
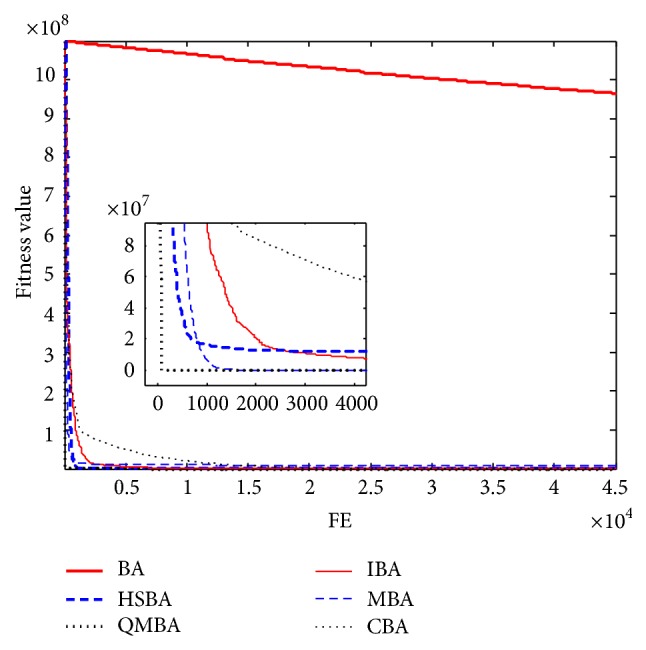
The curve of fitness value for F11.

**Figure 12 fig12:**
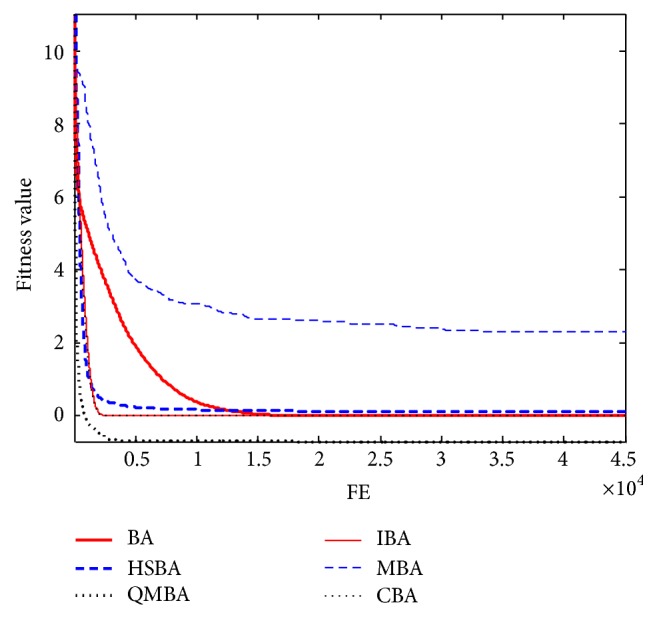
The curve of fitness value for F12.

**Figure 13 fig13:**
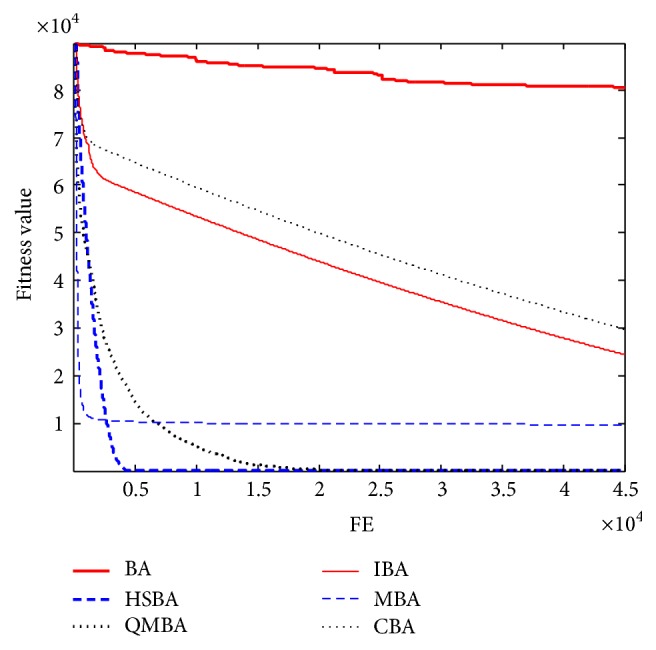
The curve of fitness value for F13.

**Figure 14 fig14:**
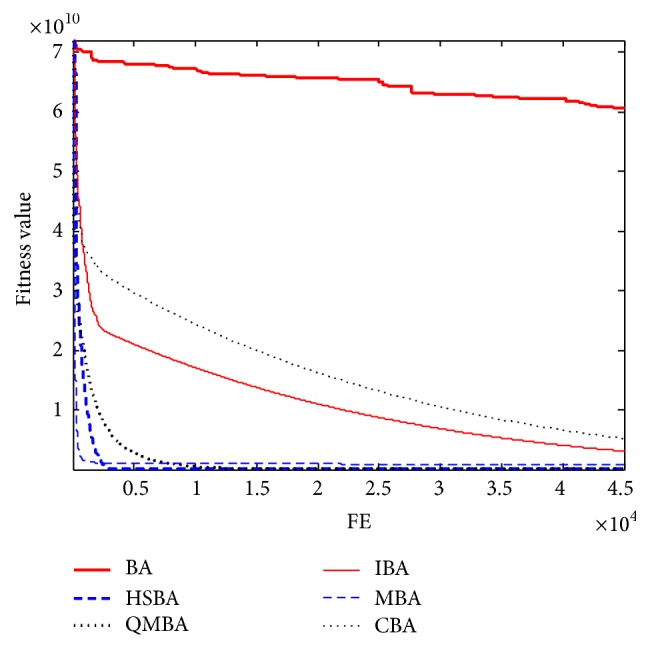
The curve of fitness value for F14.

**Figure 15 fig15:**
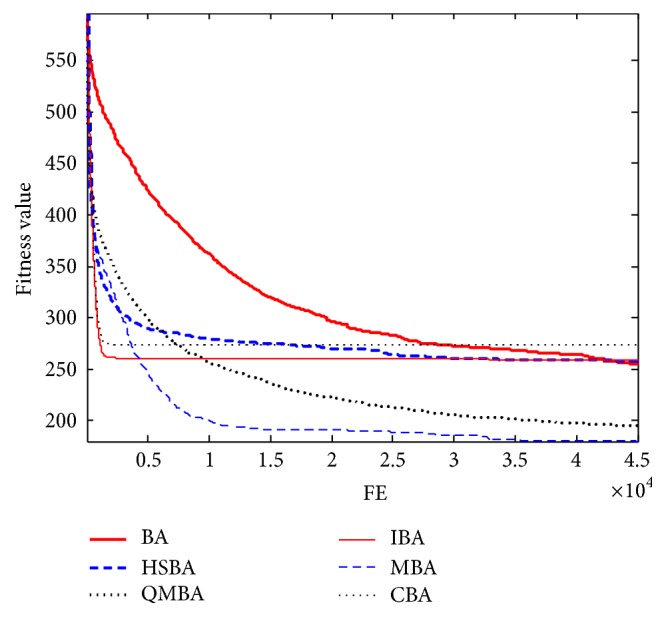
The curve of fitness value for F15.

**Figure 16 fig16:**
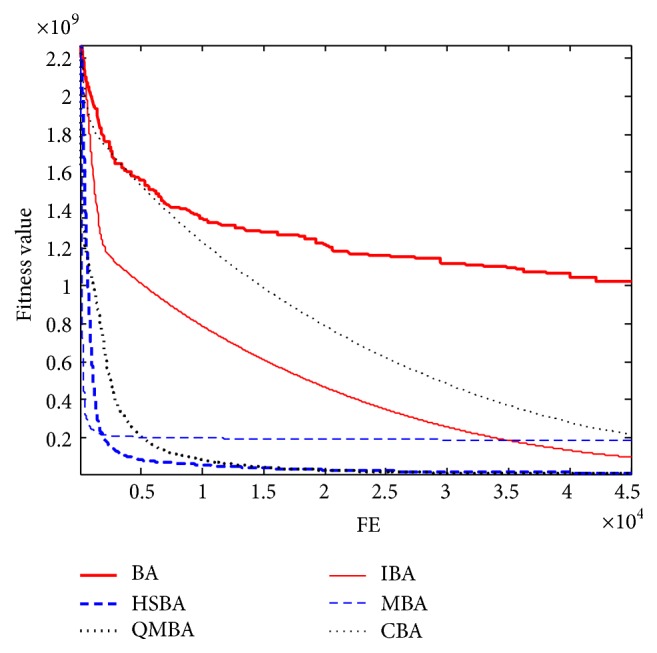
The curve of fitness value for F16.

**Figure 17 fig17:**
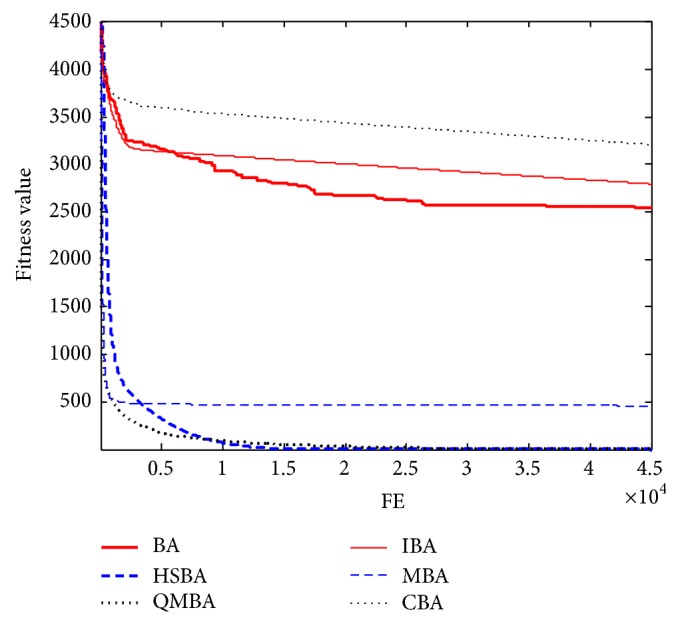
The curve of fitness value for F17.

**Figure 18 fig18:**
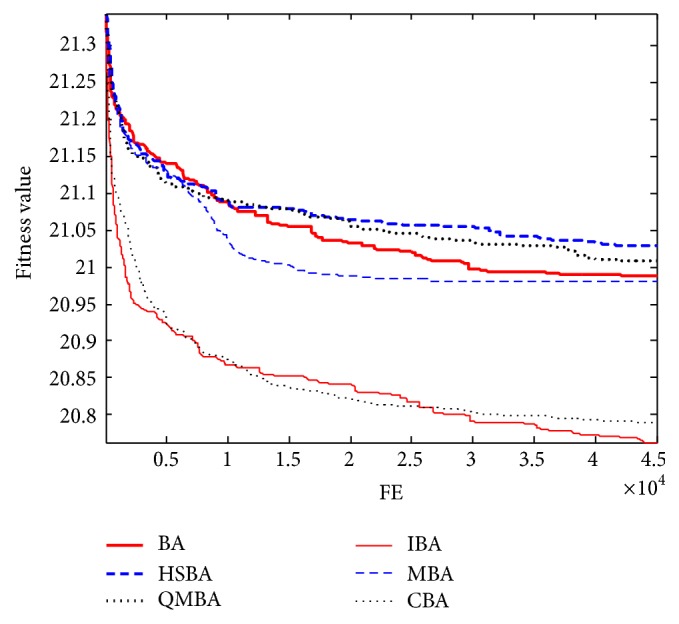
The curve of fitness value for F18.

**Figure 19 fig19:**
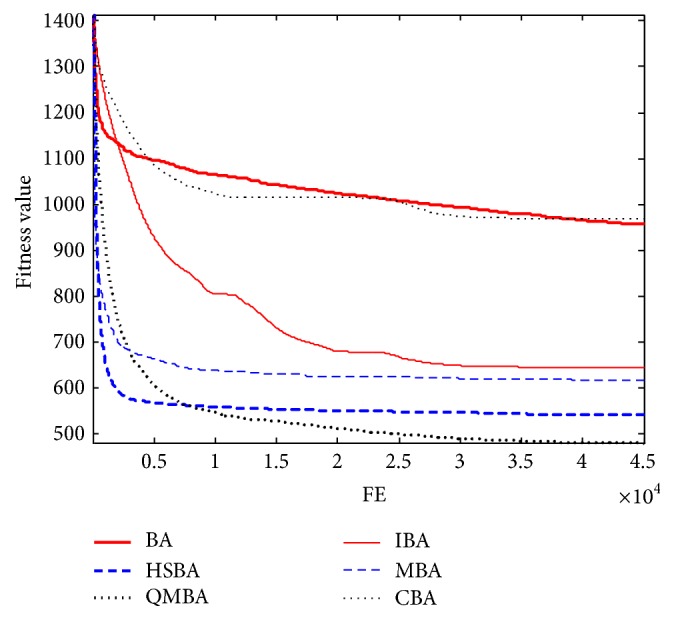
The curve of fitness value for F19.

**Figure 20 fig20:**
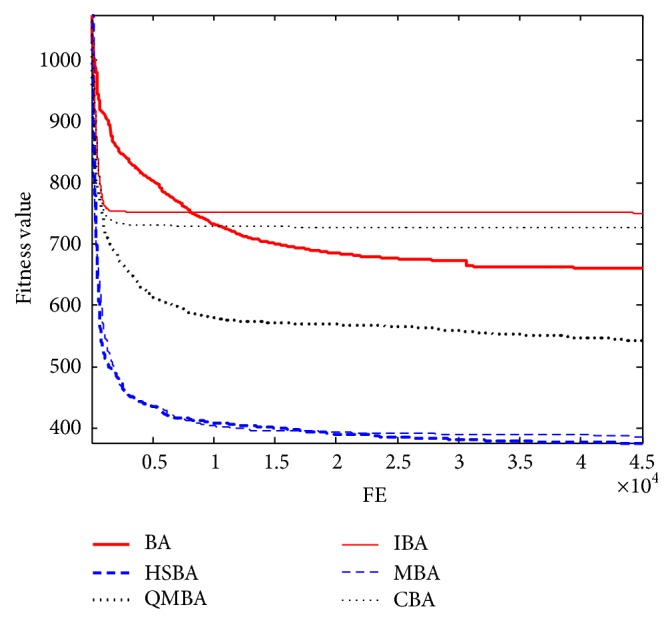
The curve of fitness value for F20.

**Figure 21 fig21:**
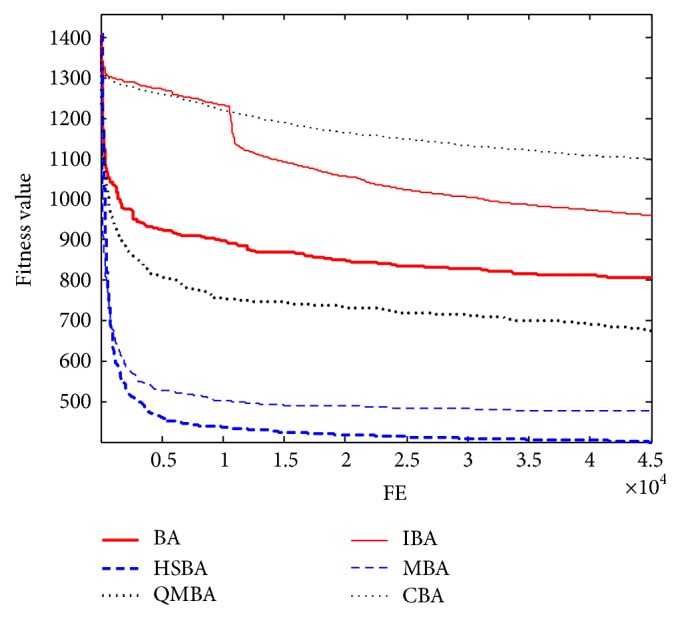
The curve of fitness value for F21.

**Figure 22 fig22:**
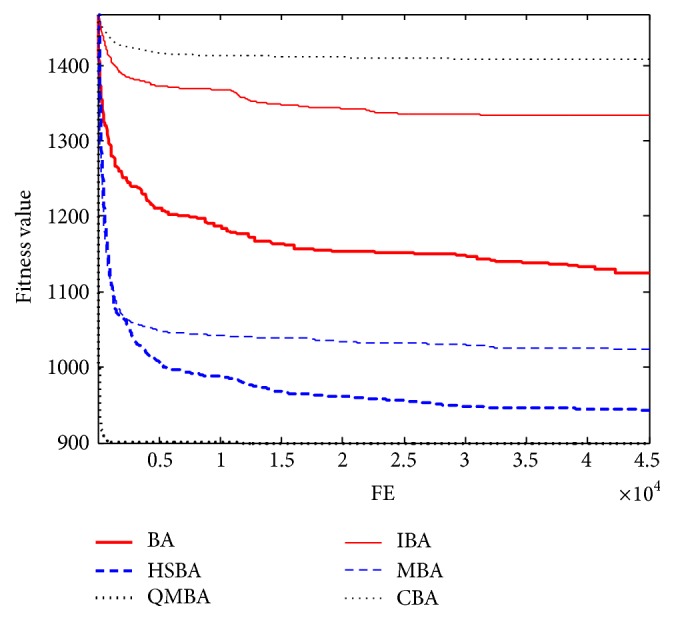
The curve of fitness value for F22.

**Figure 23 fig23:**
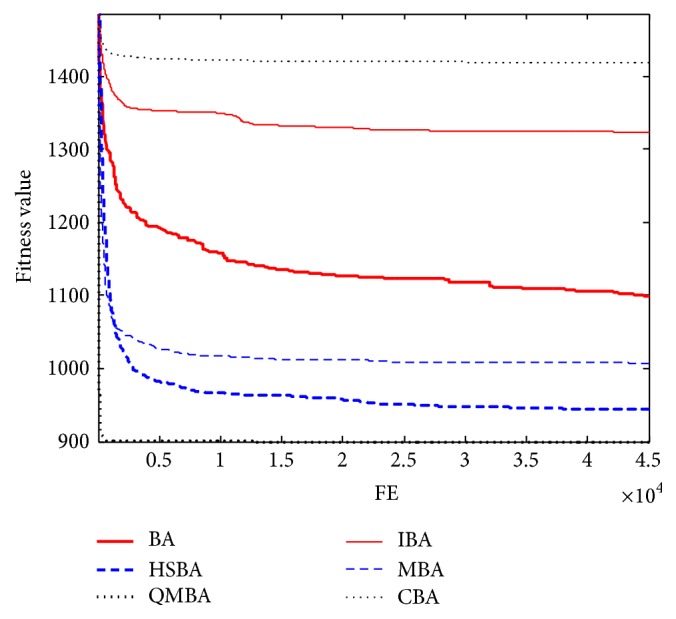
The curve of fitness value for F23.

**Figure 24:**
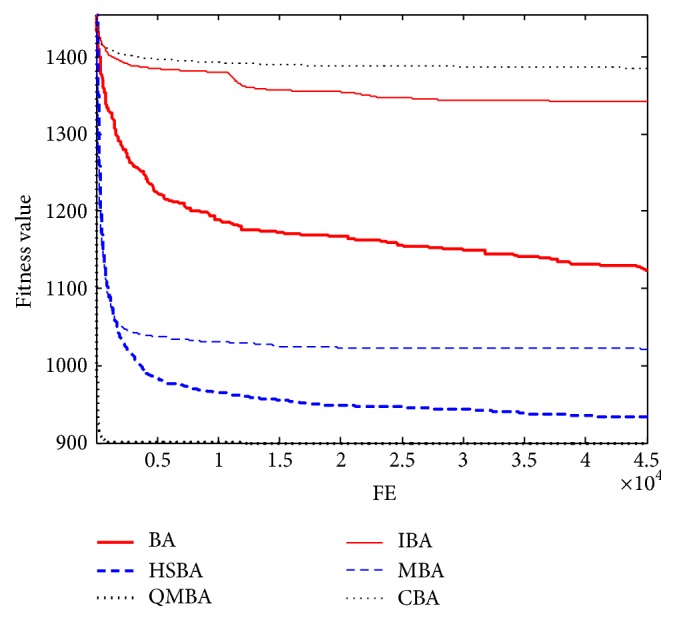
The curve of fitness value for F24.

Furthermore, the convergence graphs of BA and other variants of BA are shown in Figures 1 [Fig fig2] [Fig fig3] [Fig fig4] [Fig fig5] [Fig fig6] [Fig fig7] [Fig fig8] [Fig fig9] [Fig fig10] [Fig fig11] [Fig fig12] [Fig fig13] [Fig fig14] [Fig fig15] [Fig fig16] [Fig fig17] [Fig fig18] [Fig fig19] [Fig fig20] [Fig fig21] [Fig fig22] [Fig fig23]–24 which present the process of optimization. The fitness values shown in Figures 1 [Fig fig2] [Fig fig3] [Fig fig4] [Fig fig5] [Fig fig6] [Fig fig7] [Fig fig8] [Fig fig9] [Fig fig10] [Fig fig11] [Fig fig12] [Fig fig13] [Fig fig14] [Fig fig15] [Fig fig16] [Fig fig17] [Fig fig18] [Fig fig19] [Fig fig20] [Fig fig21] [Fig fig22] [Fig fig23]–24 are the mean objective function optimum obtained from 30 independent runs for each algorithm.  Figure 1 shows the results achieved from the six methods when Sphere Function is applied. From Figures 1, 2, 3, 4, 5, and 6, clearly, we can draw the conclusion that QMBA is significantly superior to all the other algorithms during the process of optimization, and it also has the fastest convergence rate compared with other algorithms.

Figures 7, 8, 9, 10, 11, and 12 are the curves of fitness value for multimodal functions. As can be seen from these figures, BA is more easily trapped into local minimum, especially in functions F8 and F9, and it has the slowest convergence rate; however, QMBA has also the best results and the fastest convergence rate. HSBA is showed to have the second best overall performance.

Figures 13, 14, 15, 16, 17, and 18 are the curves of fitness value for shifted rotated functions. As can be seen in these figures, CBA has faster convergence rate than other algorithms; however, it cannot obtain the better results except F15 and F18. This is probably due to CBA suffering from prematuration.

Figures 19, 20, 21, 22, 23, and 24 are the curves of fitness value hybrid composite functions. From these figures, we can observe that QMBA has the fastest convergence rate compared to other algorithms in four of six functions, while IBA and CBA severely suffer from premature convergence problem in the complex cases, especially in functions F22, F23, and F24.

## 5. Conclusions

In this paper, a variant of novel bat algorithm, namely, QMBA, is proposed by introducing quantum-behaved bat with the direction of mean best position during the searching process. In QMBA, the position of each bat not only depends on the current optimal solution, but also is determined by the mean best position with the iteration proceeding. In the process of searching, quantum behavior of bats is introduced to increase the diversity of population and avoid all bats getting trapped into local minimum. In other words, the current optimal solution leads global search to guarantee that all the bats converge, while the quantum-behaved bat and mean best position lead local search to jump out of local positions.

This new method can also speed up the global convergence rate without losing the strong robustness of the basic BA. Twenty-four benchmark test functions are tested, and compared with other variant bat algorithms for numerical optimization, from the analysis of the simulation results, we observed that the proposed QMBA makes good use of the information in past solutions more effectively to generate better quality solutions frequently and when compared to the other variants of BA showed that this approach is simple and efficient and can achieve a more accurate solution.

Our future work will focus on applying the QMBA algorithm to engineering optimization problems and developing new metahybrid approach to solve more complex optimization problem.

## Figures and Tables

**Algorithm 1:**
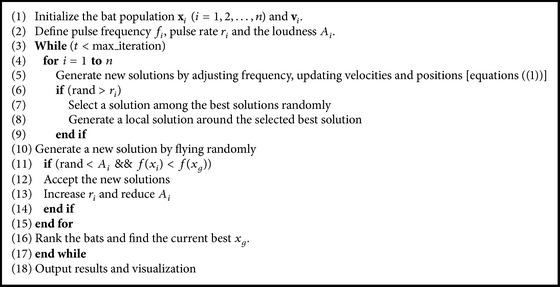
Bat algorithm (BA) pseudocode.

**Algorithm 2:**
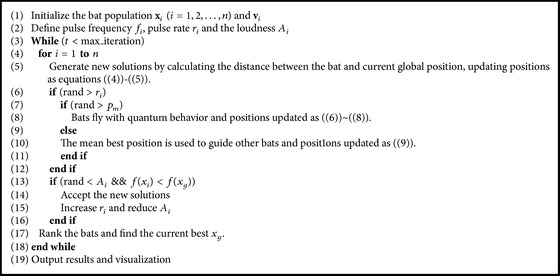
Bat algorithm with mean best position (QMBA) pseudocode.

**Table 1:** Benchmark function.

Category	Number	Function	*D*	Range	*f*_min_
I	F1	$f\left(x\right)=\overset{n}{\underset{i=1}{\sum }}x_{i}^{2}$	30	[−5.12, 5.12]	0
	F2	$f\left(x\right)=\overset{n}{\underset{i=1}{\sum }}\left|x_{i}\right|+\prod_{i=1}^{n}\left|x_{i}\right|$	30	[−10, 10]	0
	F3	$f\left(x\right)=\overset{n}{\underset{i=1}{\sum }}{\left(\overset{i}{\underset{j=1}{\sum }}x_{j}\right)}^{2}$	30	[−100, 100]	0
	F4	$f\left(x\right)=\underset{i}{\max}\left\{\left|x_{i}\right|,\;\;1\leq i\leq n\right\}$	30	[−100, 100]	0
	F5	$f\left(x\right)=\overset{n-1}{\underset{i=1}{\sum }}\left[100{\left(x_{i+1}-x_{i}\right)}^{2}+{\left(x_{i}-1\right)}^{2}\right]$	30	[−30, 30]	0
	F6	$f\left(x\right)=\overset{n}{\underset{i=1}{\sum }}ix_{i}^{4}+random\left(0,1\right)$	30	[−1.28, 1.28]	0

II	F7	$f\left(x\right)=\overset{n}{\underset{i=1}{\sum }}\left[x_{i}^{2}-10\cos\left(2\pi x_{i}\right)+10\right]$	30	[−5.12, 5.12]	0
	F8	$f\left(x\right)=-20\exp\left(-0.2\sqrt{\frac{1}{n}\overset{n}{\underset{i=1}{\sum }}x_{i}^{2}}\right)-\exp\left(\frac{1}{n}\overset{n}{\underset{i=1}{\sum }}\cos\left(2\pi x_{i}\right)\right)+20+e$	30	[−32, 32]	0
	F9	$f\left(x\right)=\frac{1}{4000}\overset{n}{\underset{i=1}{\sum }}x_{i}^{2}-\prod_{i=1}^{n}\cos\left(\frac{x_{i}}{\sqrt{i}}\right)+1$	30	[−600, 600]	0
	F10	$f\left(x\right)=\frac{\pi }{n}\left\{10\;\sin^{2}\left(\pi y_{1}\right)+\overset{n-1}{\underset{i=1}{\sum }}{\left(y_{i}-1\right)}^{2}\left[1+10\sin\left(\pi y_{i+1}\right)\right]+{\left(y_{n}-1\right)}^{2}\right\}+\overset{n}{\underset{i=1}{\sum }}u\left(x_{i},10,100,4\right)$	30	[−50, 50]	0
	F11	$f\left(x\right)=0.1\left\{\sin^{2}\left(3\pi x_{1}\right)+\overset{n-1}{\underset{i=1}{\sum }}\left[1+\sin^{2}\left(3\pi x_{i}+1\right)\right]+(x_{n}-1{)}^{2}\left[1+\sin^{2}\left(2\pi x_{n}\right)\right]\right\}+\overset{n}{\underset{i=1}{\sum }}u\left(x_{i},5,100,4\right)$	30	[−50, 50]	0
	F12	$f\left(x\right)=\left\{\left[\overset{n}{\underset{i=1}{\sum }}\;\sin^{2}\left(x_{i}\right)\right]\right.-\left.exp\left(-\overset{n}{\underset{i=1}{\sum }}x_{i}^{2}\right)\right\}*\exp\left[-\overset{n}{\underset{i=1}{\sum }}\;\sin^{2}\sqrt{\left|x_{i}\right|}\right]$	30	[−10, 10]	−1

III	F13	$f\left(x\right)=\overset{n}{\underset{i=1}{\sum }}z_{i}$	30	[−100, 100]	0
	F14	$f\left(x\right)=\overset{n}{\underset{i=1}{\sum }}\left[100{\left(z_{i}^{2}-z_{i+1}\right)}^{2}+{\left(z_{i}-1\right)}^{2}\right]$	30	[−100, 100]	0
	F15	$f\left(x\right)=\overset{n}{\underset{i=1}{\sum }}\left[z_{i}^{2}-10\cos\;(2\pi z_{i})+10\right]$	30	[−5, 5]	0
	F16	$f\left(x\right)=\overset{n}{\underset{i=1}{\sum }}{\left(10^{6}\right)}^{\left(i-1\right)/\left(D-1\right)}z_{i}^{2}$	30	[−100, 100]	0
	F17	$f\left(x\right)=\frac{1}{4000}\overset{n}{\underset{i=1}{\sum }}z_{i}^{2}-\prod_{i=1}^{n}\cos\left(\frac{z_{i}}{\sqrt{i}}\right)+1$	30	[0, 600]	0
	F18	$f\left(x\right)=-20\exp\left(-0.2\sqrt{\frac{1}{n}\overset{n}{\underset{i=1}{\sum }}z_{i}^{2}}\right)-\exp\left(\frac{1}{n}\overset{n}{\underset{i=1}{\sum }}\cos\left(2\pi z_{i}\right)\right)+20+e$	30	[−32, 32]	0

IV	F19 (CF1)	Hybrid Composition Function *f*_1_, *f*_2_ = Rastrigin's Function *f*_3_, *f*_4_ = Weierstrass Function *f*_5_, *f*_6_ = Griewank's Function *f*_7_, *f*_8_ = Ackley's Function *f*_9_, *f*_10_ = Sphere Function [*σ*_1_, *σ*_2_, *σ*_3_,…, *σ*_10_] = [1,1, 1,…, 1] [*λ*_1_, *λ*_2_, *λ*_3_,…, *λ*_10_] = [1,1, 10,10,5/60,5/60,5/32,5/32,5/100,5/100]	30	[−5, 5]	0
	F20 (CF2)	Rotated version of Hybrid Composition Function F19 Except *M*_*i*_ which are different linear transformation matrixes with condition number of 2, all other settings are the same as F19	30	[−5, 5]	0
	F21 (CF3)	F20 with noise in fitness Let F20 be *G*(*x*); then *F*_21_(*x*) = *G*(*x*)*∗*(1 + 0.2|*N*(0,1)|) All settings are the same as F20	30	[−5, 5]	0
	F22 (CF4)	Rotated Hybrid Composition Function *f*_1_, *f*_2_ = Ackley's Function *f*_3_, *f*_4_ = Rastrigin's Function *f*_5_, *f*_6_ = Sphere Function *f*_7_, *f*_8_ = Weierstrass Function *a* = 0.5, *b* = 0.3, *k*_max_ = 20 *f*_9_, *f*_10_ = Griewank's Function [*σ*_1_, *σ*_2_, *σ*_3_,…, *σ*_10_] = [1,2, 1.5,1.5,1, 1,1.5,1.5,2, 2] ***λ*** = [2*∗*5/32,2*∗*5/32,2*∗*1,1, 2*∗*5/100,5/100,2*∗*10,10, 2*∗*5/60,5/60] *M*_*i*_ are all rotation matrixes. Condition numbers are $\left[\begin{array}{cccccccccc} 2 & 3 & 2 & 3 & 2 & 3 & 20 & 30 & 200 & 300 \end{array}\right]$ **o**_10_ = [0,0,…, 0]	30	[−5, 5]	0
	F23 (CF5)	Rotated Hybrid Composition Function with narrow basin global optimum All settings are the same as F22 except [*σ*_1_, *σ*_2_, *σ*_3_,…, *σ*_10_] = [0.1,2, 1.5,1, 1,1.5,1.5,2, 2] ***λ*** = [2*∗*5/32,2*∗*5/32,2*∗*1,1, 2*∗*5/100,5/100,2*∗*10,10, 2*∗*5/60,5/60] **o**_10_ = [1,0,…, 0]	30	[−5, 5]	0
	F24 (CF6)	Rotated Hybrid Composition Function with global optimum on the bounds All settings are the same as F23 except, after loading the data file, set *o*_1(2,*j*)_ = 5, for *j* = 1,2,…, *D*/2	30	[−5, 5]	0

**Table 2:** The parameter settings of these algorithms.

Algorithms	Parameter design
BA	*f*_min_ = 0, *f*_max_ = 2, *A* = 0.5, *r* = 0.5, *α* = 0.95, *γ* = 0.05
IBA	*f*_min_ = 0, *f*_max_ = 1, *α* = *γ* = 0.9, limit = 200, *w*_min_ = 0.2, *w*_max_ = 0.9
MBA	*A* = 1.8, *r* = 0.8, *α* = *γ* = 0.9
HSBA	*A* = 0.95, *r* = 0.6, *ε* = 0.1, PAR = 0.1, HCMR = 0.95, bw = 0.5, Keep = 2
CBA	*A* and *r* are initialized by chaotic maps; *α* = *γ* = 0.9
QMBA	*A* = 0.25, *r* = 0.5, *α* = *γ* = 0.9, TH = 0.005

**Table 3:** Experimental results of unimodal benchmark functions by algorithms (best results in bold).

Algorithms		F1	F2	F3	F4	F5	F6
BA	Mean	1.0520*e* + 02	2.2066*e* + 10	6.9423*e* + 04	8.7571*e* + 01	2.1222*e* + 08	1.7977*e* + 01
	Min	8.4269*e* + 01	1.0492*e* + 02	4.0504*e* + 04	8.7084*e* + 01	1.8225*e* + 08	3.4554*e* + 00
	Max	1.6958*e* + 02	2.6524*e* + 11	8.3920*e* + 04	8.9196*e* + 01	2.4983*e* + 08	8.6022*e* + 01
	Std	2.6294*e* + 01	6.1313*e* + 10	1.2163*e* + 04	4.8092*e* − 01	1.6327*e* + 07	2.8488*e* + 01

IBA	Mean	1.3493*e* − 03	1.2324*e* + 01	1.9501*e* + 03	2.3259*e* + 01	1.0208*e* + 02	3.7488*e* − 02
	Min	1.1048*e* − 03	2.2161*e* − 01	2.0172*e* + 02	1.5919*e* + 01	2.2386*e* + 01	2.0379*e* − 02
	Max	1.7220*e* − 03	1.0659*e* + 02	5.8598*e* + 03	3.3558*e* + 01	5.8034*e* + 02	6.1803*e* − 02
	Std	1.6313*e* − 04	2.5659*e* + 01	1.4319*e* + 03	4.5757*e* + 00	1.4067*e* + 02	1.1157*e* − 02

MBA	Mean	2.2972*e* + 01	4.0940*e* + 01	2.4648*e* + 04	4.7065*e* + 01	5.5687*e* + 06	4.6924*e* + 00
	Min	1.1901*e* + 01	2.9348*e* + 01	1.6771*e* + 04	3.3576*e* + 01	1.1137*e* + 06	2.2631*e* + 00
	Max	3.4470*e* + 01	5.0545*e* + 01	3.2960*e* + 04	5.6452*e* + 01	1.5316*e* + 07	9.3272*e* + 00
	Std	5.9513*e* + 00	5.3719*e* + 00	3.9012*e* + 03	5.5703*e* + 00	3.1130*e* + 06	1.7066*e* + 00

HSBA	Mean	1.4556*e* − 01	1.6079*e* + 00	4.0125*e* + 00	1.1026*e* + 00	6.4326*e* + 01	1.3798*e* − 01
	Min	8.6816*e* − 02	1.0869*e* + 00	9.7708*e* − 01	1.6676*e* − 01	3.8138*e* + 01	5.0585*e* − 02
	Max	1.9280*e* − 01	1.9813*e* + 00	6.9179*e* + 00	6.3851*e* + 00	1.5983*e* + 02	2.4781*e* − 01
	Std	2.9025*e* − 02	2.0790*e* − 01	1.2881*e* + 00	1.5226*e* + 00	3.4549*e* + 01	5.0364*e* − 02

CBA	Mean	1.3400*e* − 03	1.1039*e* + 05	7.4202*e* + 03	3.2386*e* + 01	1.1515*e* + 02	3.5127*e* − 02
	Min	9.5933*e* − 04	7.8449*e* + 00	1.6956*e* + 03	2.1754*e* + 01	2.3036*e* + 01	1.2743*e* − 02
	Max	1.6616*e* − 03	2.7800*e* + 06	1.8489*e* + 04	4.9108*e* + 01	4.2422*e* + 02	6.8828*e* − 02
	Std	1.6009*e* − 04	4.9947*e* + 05	3.6881*e* + 03	7.6207*e* + 00	1.3531*e* + 02	1.3576*e* − 02

QMBA	Mean	**7.6898e − 70**	**6.1368e − 36**	**1.7587e − 21**	**5.1883e − 35**	**2.3768e + 01**	**4.2512e − 04**
	Min	**3.3739e − 84**	**7.3443e − 41**	**2.2692e − 39**	**1.7424e − 39**	**0.0000e + 00**	**4.2318e − 05**
	Max	**2.1833e − 68**	**1.4449e − 34**	**4.3799e − 20**	**1.1658e − 33**	**2.7561e + 01**	**1.1393e − 03**
	Std	**3.9168e − 69**	**2.5906e − 35**	**7.8785e − 21**	**2.1000e − 34**	**7.9530e + 00**	**3.1788e − 04**

**Table 4:** *p* values calculated for *t*-test on unimodal benchmark functions.

Algorithms	F1	F2	F3	F4	F5	F6
BA	2.317*e* − 29	5.748*e* − 02	1.367*e* − 37	4.476*e* − 124	9.919*e* − 58	1.232*e* − 03
IBA	1.489*e* − 46	1.223*e* − 03	8.049*e* − 10	7.398*e* − 35	4.053*e* − 03	2.840*e* − 25
MBA	1.465*e* − 28	1.485*e* − 44	5.116*e* − 40	4.473*e* − 47	1.217*e* − 13	2.319*e* − 21
HSBA	1.532*e* − 34	6.517*e* − 45	6.411*e* − 24	2.527*e* − 04	7.394*e* − 08	3.149*e* − 21
CBA	7.616*e* − 47	2.388*e* − 03	1.463*e* − 15	1.006*e* − 30	5.995*e* − 04	6.406*e* − 20

**Table 5:** Experimental results of multimodal benchmark functions by algorithms (best results in bold).

Algorithms		F7	F8	F9	F10	F11	F12
BA	Mean	2.2196*e* + 02	1.9930*e* + 01	5.3680*e* + 02	4.2534*e* + 08	9.6509*e* + 08	2.2439*e* − 03
	Min	9.8765*e* + 01	1.9905*e* + 01	4.2285*e* + 02	3.9948*e* + 08	8.4923*e* + 08	1.5573*e* − 03
	Max	3.9100*e* + 02	1.9946*e* + 01	5.5131*e* + 02	4.9814*e* + 08	1.0976*e* + 09	3.3311*e* − 03
	Std	8.8763*e* + 01	8.6821*e* − 03	3.1930*e* + 01	2.6578*e* + 07	5.3834*e* + 07	4.2665*e* − 04

IBA	Mean	1.4073*e* + 02	1.4867*e* + 01	6.3576*e* + 01	1.7971*e* + 01	8.9507*e* + 01	1.3564*e* − 03
	Min	9.7746*e* + 01	1.2459*e* + 01	3.2813*e* + 01	8.0753*e* + 00	6.5495*e* + 01	1.0861*e* − 03
	Max	1.9825*e* + 02	1.7726*e* + 01	9.7849*e* + 01	3.0759*e* + 01	1.1203*e* + 02	1.6601*e* − 03
	Std	3.8512*e* + 01	1.3673*e* + 00	1.6637*e* + 01	5.4157*e* + 00	1.2749*e* + 01	1.4210*e* − 04

MBA	Mean	1.5356*e* + 02	1.4910*e* + 01	8.0691*e* + 01	1.7537*e* + 06	8.5116*e* + 06	2.2243*e* + 00
	Min	1.2267*e* + 02	1.1881*e* + 01	4.8878*e* + 01	4.6576*e* + 03	9.4329*e* + 05	1.3556*e* + 00
	Max	1.8516*e* + 02	1.6365*e* + 01	1.3017*e* + 02	7.9333*e* + 06	2.4844*e* + 07	3.2322*e* + 00
	Std	1.6806*e* + 01	1.1256*e* + 00	1.8766*e* + 01	1.6657*e* + 06	5.6320*e* + 06	4.9722*e* − 01

HSBA	Mean	3.6746*e* + 01	6.3046*e* − 01	9.1292*e* − 03	1.7592*e* − 03	2.7202*e* − 02	1.0172*e* − 01
	Min	1.8996*e* + 01	3.7880*e* − 01	5.2827*e* − 03	9.4920*e* − 04	1.2030*e* − 02	−6.0711*e* − 01
	Max	6.2000*e* + 01	3.8051*e* + 00	1.3725*e* − 02	6.9317*e* − 03	3.9820*e* − 02	1.6138*e* − 01
	Std	1.0779*e* + 01	5.9347*e* − 01	2.1471*e* − 03	1.0197*e* − 03	6.1571*e* − 03	1.3415*e* − 01

CBA	Mean	1.6923*e* + 02	1.7117*e* + 01	1.2186*e* + 02	2.2994*e* + 01	9.2521*e* + 01	1.3414*e* − 03
	Min	6.3918*e* + 01	1.3699*e* + 01	5.8054*e* + 01	8.3428*e* + 00	7.3413*e* + 01	9.4095*e* − 04
	Max	1.9831*e* + 02	1.9960*e* + 01	2.0184*e* + 02	3.6968*e* + 01	1.1491*e* + 02	1.6389*e* − 03
	Std	4.4037*e* + 01	1.6093*e* + 00	3.8238*e* + 01	7.2960*e* + 00	1.0007*e* + 01	1.7965*e* − 04

QMBA	Mean	**0.0000e + 00**	**8.8818e − 16**	**0.0000e + 00**	**1.5705e − 32**	**1.3498e − 32**	**−8.3333e − 01**
	Min	**0.0000e + 00**	**8.8818e − 16**	**0.0000e + 00**	**1.5705e − 32**	**1.3498e − 32**	**−1.0000e + 00**
	Max	**0.0000e + 00**	**8.8818e − 16**	**0.0000e + 00**	**1.5705e − 32**	**1.3498e − 32**	**1.7997e − 30**
	Std	**0.0000e + 00**	**0.0000e + 00**	**0.0000e + 00**	**5.4738e − 48**	**5.4738e − 48**	**2.2439e − 03**

**Table 6:** *p* values calculated for *t*-test on multimodal benchmark functions.

Algorithms	F7	F8	F9	F10	F11	F12
BA	1.679*e* − 19	6.569*e* − 188	3.765*e* − 64	6.420*e* − 63	9.280*e* − 66	1.828*e* − 17
IBA	2.390*e* − 27	2.701*e* − 53	2.451*e* − 28	2.936*e* − 25	1.462*e* − 42	1.910*e* − 17
MBA	5.346*e* − 49	3.351*e* − 58	5.435*e* − 31	4.746*e* − 07	3.590*e* − 11	4.241*e* − 34
HSBA	7.736*e* − 26	3.915*e* − 07	9.787*e* − 31	4.412*e* − 13	1.311*e* − 31	2.058*e* − 18
CBA	1.841*e* − 28	9.488*e* − 53	2.122*e* − 24	3.641*e* − 24	2.745*e* − 49	1.912*e* − 17

**Table 7:** Experimental results of shifted and rotated benchmark functions by algorithms (best results in bold).

Algorithms		F13	F14	F15	F16	F17	F18
BA	Mean	8.0602*e* + 04	6.0696*e* + 10	2.5512*e* + 02	1.0210*e* + 09	2.5458*e* + 03	2.0988*e* + 01
	Min	6.1153*e* + 04	3.0545*e* + 10	1.5947*e* + 02	6.1482*e* + 08	1.1580*e* + 03	2.0797*e* + 01
	Max	8.7793*e* + 04	7.1715*e* + 10	5.3011*e* + 02	1.5343*e* + 09	3.6974*e* + 03	2.1122*e* + 01
	Std	7.9571*e* + 03	1.2295*e* + 10	8.5965*e* + 01	2.1564*e* + 08	6.2572*e* + 02	7.3264*e* − 02

IBA	Mean	2.4479*e* + 04	3.0626*e* + 09	2.5975*e* + 02	9.6466*e* + 07	2.7969*e* + 03	**2.0763e + 01**
	Min	1.9079*e* + 04	1.8150*e* + 09	1.8629*e* + 02	4.2226*e* + 07	2.3800*e* + 03	**2.0563e + 01**
	Max	3.4241*e* + 04	5.0137*e* + 09	3.3371*e* + 02	1.4886*e* + 08	3.3734*e* + 03	**2.0916e + 01**
	Std	3.4561*e* + 03	7.8361*e* + 08	3.9198*e* + 01	2.9739*e* + 07	2.1945*e* + 02	**1.0013e − 01**

MBA	Mean	9.4981*e* + 03	9.0058*e* + 08	**1.8004e + 02**	1.8433*e* + 08	4.5504*e* + 02	2.0980*e* + 01
	Min	4.3322*e* + 03	2.4050*e* + 08	1.2816*e* + 02	5.0917*e* + 07	2.2488*e* + 02	2.0828*e* + 01
	Max	1.5707*e* + 04	3.0371*e* + 09	**2.2829e + 02**	3.2034*e* + 08	7.3538*e* + 02	2.1086*e* + 01
	Std	3.0561*e* + 03	5.2168*e* + 08	**2.6217e + 01**	7.1234*e* + 07	1.3193*e* + 02	6.3286*e* − 02

HSBA	Mean	**1.8143e − 01**	3.9316*e* + 03	2.5801*e* + 02	1.4158*e* + 07	**1.2396e − 01**	2.1028*e* + 01
	Min	1.1470*e* − 01	**1.4240e + 02**	1.8963*e* + 02	6.2258*e* + 06	**6.1688e − 02**	2.0814*e* + 01
	Max	**2.4444e − 01**	**7.7746e + 03**	3.3713*e* + 02	4.6223*e* + 07	**2.5443e − 01**	2.1106*e* + 01
	Std	**2.9407e − 02**	**2.1534e + 03**	3.4819*e* + 01	8.9814*e* + 06	**3.8659e − 02**	6.3454*e* − 02

CBA	Mean	2.9796*e* + 04	5.1498*e* + 09	2.7382*e* + 02	2.1605*e* + 08	3.2119*e* + 03	2.0789*e* + 01
	Min	2.1297*e* + 04	2.1656*e* + 09	1.9228*e* + 02	8.7480*e* + 07	2.4267*e* + 03	2.0628*e* + 01
	Max	3.7477*e* + 04	1.0308*e* + 10	3.3493*e* + 02	4.2612*e* + 08	3.8657*e* + 03	2.0942*e* + 01
	Std	4.2828*e* + 03	2.0642*e* + 09	3.8427*e* + 01	8.9164*e* + 07	3.8695*e* + 02	7.9736*e* − 02

QMBA	Mean	3.5374*e* − 01	**3.6205e + 03**	1.9473*e* + 02	**8.5901e + 06**	2.1283*e* + 00	2.1008*e* + 01
	Min	**1.1148e − 01**	2.6836*e* + 02	**7.8661e + 01**	**3.6992e + 06**	1.0633*e* + 00	2.0885*e* + 01
	Max	1.0003*e* + 00	1.1367*e* + 04	2.5432*e* + 02	**1.3569e + 07**	4.6080*e* + 00	2.1097*e* + 01
	Std	2.3888*e* − 01	3.2243*e* + 03	3.6201*e* + 01	**2.6158e + 06**	9.5384*e* − 01	5.1029*e* − 02

**Table 8:** *p* values calculated for *t*-test on shifted and rotated benchmark functions.

Algorithms	F13	F14	F15	F16	F17	F18
BA	1.532*e* − 51	3.562*e* − 34	9.394*e* − 04	5.246*e* − 33	1.016*e* − 29	2.304*e* − 01
IBA	8.960*e* − 43	7.742*e* − 29	1.588*e* − 08	9.590*e* − 23	3.212*e* − 57	5.906*e* − 17
MBA	7.156*e* − 24	4.317*e* − 13	8.200*e* − 02	3.125*e* − 19	5.487*e* − 26	7.426*e* − 02
HSBA	2.918*e* − 02	6.673*e* − 01	6.727*e* − 09	2.194*e* − 03	2.696*e* − 16	1.816*e* − 01
CBA	2.432*e* − 42	1.857*e* − 19	4.712*e* − 11	3.896*e* − 18	1.265*e* − 46	5.559*e* − 18

**Table 9:** Experimental results of hybrid composite benchmark functions by algorithms (best results in bold).

Algorithms		F19	F20	F21	F22	F23	F24
BA	Mean	9.5618*e* + 02	6.6067*e* + 02	8.0640*e* + 02	1.1242*e* + 03	1.0997*e* + 03	1.1241*e* + 03
	Min	6.1358*e* + 02	3.7942*e* + 02	5.5352*e* + 02	1.0306*e* + 03	9.8772*e* + 02	1.0554*e* + 03
	Max	1.1205*e* + 03	8.9018*e* + 02	1.0230*e* + 03	1.2059*e* + 03	1.2254*e* + 03	1.2457*e* + 03
	Std	1.0641*e* + 02	1.1969*e* + 02	1.4405*e* + 02	5.6971*e* + 01	5.3599*e* + 01	5.3205*e* + 01

IBA	Mean	6.4540*e* + 02	7.5060*e* + 02	9.6044*e* + 02	1.3335*e* + 03	1.3238*e* + 03	1.3419*e* + 03
	Min	4.0066*e* + 02	7.0373*e* + 02	6.2563*e* + 02	1.2690*e* + 03	1.2388*e* + 03	1.2700*e* + 03
	Max	1.1696*e* + 03	7.9351*e* + 02	1.2650*e* + 03	1.3964*e* + 03	1.3828*e* + 03	1.3951*e* + 03
	Std	3.3213*e* + 02	2.2954*e* + 01	1.6472*e* + 02	3.5586*e* + 01	3.7108*e* + 01	3.3787*e* + 01

MBA	Mean	6.1633*e* + 02	3.8661*e* + 02	4.7736*e* + 02	1.0240*e* + 03	1.0072*e* + 03	1.0218*e* + 03
	Min	5.3347*e* + 02	3.1116*e* + 02	3.1559*e* + 02	9.8563*e* + 02	9.6854*e* + 02	9.7411*e* + 02
	Max	8.0680*e* + 02	4.6281*e* + 02	6.9084*e* + 02	1.0607*e* + 03	1.0587*e* + 03	1.0681*e* + 03
	Std	7.5831*e* + 01	3.3274*e* + 01	1.1163*e* + 02	2.1509*e* + 01	2.4038*e* + 01	2.4225*e* + 01

HSBA	Mean	5.4040*e* + 02	**3.7639e + 02**	**4.0158e + 02**	9.4292*e* + 02	9.4360*e* + 02	9.3351*e* + 02
	Min	4.4617*e* + 02	**2.3980e + 02**	**2.4471e + 02**	9.1859*e* + 02	9.1968*e* + 02	9.1525*e* + 02
	Max	1.0346*e* + 03	**5.1007e + 02**	**5.6114e + 02**	9.8567*e* + 02	1.0001*e* + 03	9.8476*e* + 02
	Std	1.7834*e* + 02	**6.3846e + 01**	**7.5353e + 01**	2.0442*e* + 01	1.8972*e* + 01	1.6070*e* + 01

CBA	Mean	9.6917*e* + 02	7.2755*e* + 02	1.1000*e* + 03	1.4076*e* + 03	1.4183*e* + 03	1.3862*e* + 03
	Min	4.0076*e* + 02	6.3728*e* + 02	9.8843*e* + 02	1.3586*e* + 03	1.3625*e* + 03	1.3639*e* + 03
	Max	1.3061*e* + 03	7.7493*e* + 02	1.3919*e* + 03	1.4419*e* + 03	1.4468*e* + 03	1.4186*e* + 03
	Std	3.7487*e* + 02	3.5249*e* + 01	1.0808*e* + 02	1.8473*e* + 01	2.0678*e* + 01	1.3209*e* + 01

QMBA	Mean	**4.7921e + 02**	5.4202*e* + 02	6.7557*e* + 02	**9.0000e + 02**	**9.0000e + 02**	**9.0000e + 02**
	Min	**4.0309e + 02**	3.8418*e* + 02	4.3168*e* + 02	**9.0000e + 02**	**9.0000e + 02**	**9.0000e + 02**
	Max	**7.9571e + 02**	6.4515*e* + 02	8.2616*e* + 02	**9.0000e + 02**	**9.0000e + 02**	**9.0000e + 02**
	Std	**1.1200e + 02**	6.9503*e* + 01	1.0464*e* + 02	**0.0000e + 00**	**0.0000e + 00**	**0.0000e + 00**

**Table 10:** *p* values calculated for *t*-test on hybrid benchmark functions.

Algorithms	F19	F20	F21	F22	F23	F24
BA	4.911*e* − 16	6.116*e* − 04	2.751*e* − 03	1.735*e* − 19	1.086*e* − 18	1.738*e* − 20
IBA	4.562*e* − 02	5.961*e* − 15	1.812*e* − 07	2.907*e* − 37	3.253*e* − 36	2.028*e* − 38
MBA	7.994*e* − 05	1.078*e* − 10	1.737*e* − 06	2.807*e* − 25	2.453*e* − 21	3.648*e* − 23
HSBA	2.130*e* − 01	3.303*e* − 09	2.753*e* − 11	3.778*e* − 11	3.247*e* − 12	4.543*e* − 11
CBA	3.144*e* − 06	1.207*e* − 12	8.094*e* − 15	1.336*e* − 50	4.355*e* − 49	2.048*e* − 55
